# Similarities and Differences between Exome Sequences Found in a Variety of Tissues from the Same Individual

**DOI:** 10.1371/journal.pone.0101412

**Published:** 2014-07-01

**Authors:** Alberto Gómez-Ramos, Rafael Sanchez-Sanchez, Ashraf Muhaisen, Alberto Rábano, Eduardo Soriano, Jesús Avila

**Affiliations:** 1 Centro de Biología Molecular "Severo Ochoa" CSIC-UAM, Madrid, Spain; 2 Hospital Universitario Reina Sofía, IMIBIC, Córdoba, Spain; 3 Fundación CIEN, Vallecas, Spain; 4 Department of Cell Biology, University of Barcelona, Barcelona, Spain; 5 Institut de Recerca de l'Hospital Universitari de la Vall d'Hebron (VHIR), Barcelona, Spain; 6 Centro de Investigación Biomédica en Red de Enfermedades Neurodegenerativas (CIBERNED), Madrid, Spain; IIBB/CSIC/IDIBAPS, Spain

## Abstract

DNA is the most stable nucleic acid and most important store of genetic information. DNA sequences are conserved in virtually all the cells of a multicellular organism. To analyze the sequences of various individuals with distinct pathological disorders, DNA is routinely isolated from blood, independently of the tissue that is the target of the disease. This approach has proven useful for the identification of familial diseases where mutations are present in parental germinal cells. With the capacity to compare DNA sequences from distinct tissues or cells, present technology can be used to study whether DNA sequences in tissues are invariant. Here we explored the presence of specific SNVs (Single Nucleotide Variations) in various tissues of the same individual. We tested for the presence of tissue-specific exonic SNVs, taking blood exome as a control. We analyzed the chromosomal location of these SNVs. The number of SNVs per chromosome was found not to depend on chromosome length, but mainly on the number of protein-coding genes per chromosome. Although similar but not identical patterns of chromosomal distribution of tissue-specific SNVs were found, clear differences were detected. This observation supports the notion that each tissue has a specific SNV exome signature.

## Introduction

Typical research and diagnostic practices analyze DNA from a single tissue, commonly blood. Although it has been proposed that DNA sequences show invariability [1], errors may occur in DNA processing during development, resulting in DNA sequence variants that spread to cellular lineages. The development of a human being starts from the zygote and goes on to form an organism with 10^13^ to 10^14^ cells [2]. During this process, a number of somatic mutations may take place, mainly caused by errors in DNA replication or reparation that primarily occur during cell proliferation. Once the adult organism has been formed, cell proliferation from adult stem cells may lead to the appearance of somatic mutations during adulthood, thus resulting in the formation of genetic mosaicism. In addition, somatic genomic variability may include cell lineages in various tissues [3], [4]. Accordingly, a number of DNA variations may arise during early embryonic periods, later developmental phases, or during adulthood, with their frequency and location being determined by when and where they were formed.

The cell populations of tissues differ, and a given population may show a specific SNV in its DNA. Thus, the identification of variants in a small cell population in a specific tissue calls for a DNA sequencing method with high sensitivity [5]. Sequencing DNA samples by the Sanger method is a useful and reliable approach for the detection of sequence variations, but it is designed to analyze homogeneous samples. Consequently, in a small proportion of isolated DNA of the cell population of interest, the method is not sensitive enough to detect a SNV. Moreover, this variant may not be distinguishable from signal noise in chromatograms.

Several types of variation may arise, like missense and nonsense base substitutions (SNV), deletions, and insertions, or variations caused by other mechanisms, such as the movement of transportable elements [6], [7]. Also, the likelihood of a variation in DNA sequence may differ depending on the cell origin. Indeed, some cells may be more sensitive to DNA damage than others. Furthermore, not all the bases in the human genome are equally prone to chance mutations [8]. Also, chromosome distribution inside the cell may influence the occurrence of tissue-specific exonic SNVs and their distribution in chromosomes.

Here we studied the presence and distribution of SNVs along chromosomes, with a special emphasis on the number of tissue-specific exonic SNVs and their location. We detected a particular distribution of exonic SNVs that appears to be related mainly to the number of protein-coding genes in each chromosome. We also identified tissue-specific SNVs (comparing tissue by tissue) whose distribution along the chromosomes differed in function of the tissue studied. Moreover, we observed that certain tissues have a similar pattern of SNV distribution in some chromosomes, correlating with their embryonic origin. This observation would support the notion of a common embryonic origin.

## Materials and Methods

### Nomenclature

The term single nucleotide variation (SNV) is used to define a variation in a single nucleotide that occurs in the genome, while the more specific term single nucleotide polymorphism (SNP) is understood to be a single nucleotide variation that arises at appreciable frequency (at least 1%) in the population [9]. The term tissue-specific SNV is used to define a variation found only in one of the samples analyzed when these are compared pairwise, but it could be shared with other tissues.

### Ethical statement

Samples from donors A and B, were obtained from the Spanish Brain Bank (*Banco de Tejidos CIEN* [BT-CIEN], http://bt.fundacioncien.es/) and samples from donor C were obtained from the *Biobanco del Sistema Sanitario Público de Andalucía* (http://www.juntadeandalucia.es/salud/biobanco/). Donors gave their written informed consent and the tissues were obtained using protocols approved by the ethical committee of the *Spanish Brain Bank* and the *Biobanco del Sistema Sanitario Público de Andalucía*. Our work was previously approved by the ethical committee of our center (*Comité de Ética de la Investigación conjunto CNB-CBMSO*, http://www.cnb.csic.es/~cei/).

### Origin of human samples and characteristics of donors

Blood and hippocampus samples were obtained from patients A and B, and samples of the following were obtained from patient C: adipose tissue, blood, frontal cortex, kidney, liver, lung, motor cortex, lung, skeletal muscle, skin, small intestine, spinal cord, spleen, suprarenal cortex, and testis. The characteristics of the donors are described in Table 1.

**Table 1:** Characteristics of subjects studied.

Subject	Gender	Age	Cause of Death	Other known diseases
A	M	84	Pneumonia	Diabetes Prostatic hyperplasia
B	M	46	Pneumonia	Intersticial fibrosis
C	M	66	Pneumonia	Enphysema Myocardiopathy Atherosclerosis

Subject A suffered from diabetes, and had surgery for prostate and cataract.

Subject B suffered from an amyopatic dermatomyositis with rapidly progressing interstitial lung disease. During the last phase of his disease the patient received ceftriaxone, fluconazole, meropenem, voriconazole and linezolid.

Subject C suffered from multifocal and bilateral enphysema, dilated myocardiopathy and atherosclerosis.

### Tissue sample preparation

Post mortem tissues were obtained through a rapid pathological autopsy shortly after death. The post mortem interval was 3 hours. According to the protocol, immediately after the autopsy the fresh tissues were flash-frozen in −50°C isopentane. Thereafter each frozen tissue was introduced in a −80°C freezer for long-term preservation. Specific frozen tissue samples of various brain regions were obtained from the corresponding slices after a 2 hour period of temperature soothing. Each sample was obtained with the aid of sterile disposable material and introduced in sterile cryo-tubes. Thereafter the samples were kept at −80°C. The rest of tissues were obtained and frozen with similar protocols. Blood samples were obtained simultaneously with routine blood extractions.

### DNA isolation

All genomic DNA samples were isolated from blood and the rest of the tissues using Qiagen kits (DNeasy Blood and Tissue, ref:69504), according to the manufacturer instructions.

#### Sample processing for exome sequencing

3×10^−6^ g of genomic DNA was fragmented to an average size of 200 bp using a Covaris LE220 instrument. Short insert libraries were obtained using the Illumina TruSeq DNA Sample Preparation Kit. Exonic sequences were enriched using NimbleGen Sequence Capture Human Exome 2.1 M Array. Paired-end sequences of 91 nucleotides from each end were generated using an Illumina HiSeq 2000 instrument to an average of 50× coverage. Sequences were generated in fastaq format.

#### Bioinformatics analysis

Samples were aligned to the human reference genome version hg19 [10] using the BWA aligner software [11] with default parameters. For each patient, all the samples were pre-processed using Picard software to remove duplicate reads (http://picard.sourceforge.net/). Local realignment was performed around indels to improve SNV calling in these conflictive areas (IndelRealigner from the Genome Analyzer Toolkit, GATK, version 2.1-8 [12]). Base quality scores were recalibrated using BaseRecalibrator from GATK. The UnifiedGenotyper algorithm from GATK was then used with default parameters (see [13], [14] for details) to call SNVs, and a first file including raw calls was obtained. We then separated the indels from the rest of the calls and only Single Nucleotide Variations (SNVs) were considered for the analysis. These variants were filtrated with VariantFiltration, from GATK, using the following parameters: coverage: DP >10, DP >20, DP>50 or DP >100, depending on the case of study; QD <2.0; FS >60.0, MQ <35.0; HaplotypeScore >13.0; MQRankSum <−12.5 and ReadPosRankSum <−8.0. We selected only calls that passed these filters. Variants were annotated using the dbSNP database version 135 [15], the UCSC human RefGene [16], and the software snpEFF (version 2_0_5) [17]. In order to manipulate the files containing variations and to determine how many of these variations were unique or common to different tissues, we used the software VCFtools [18]. All analyses to determine enrichments in Gene Ontologies (GOs) based on gene lists were performed using the web-based tool GeneCodis [19].

## Results

### Similar number of SNVs in blood DNA from two different subjects

As a first step, we performed exome sequencing of blood DNA from two different subjects. This procedure was performed considering a range of read depths (10 to 100). When a low read depth (10 reads) was used, a difference of about 0.1% in the SNVs of the genomic sequences of the two subjects was observed. This difference was attributed to SNVs and was in the order of the expected data [8]. Nevertheless, to achieve a deeper coverage, for the rest of the experiments we considered only SNVs observed in at least 20 reads. More than 98% of the SNVs obtained for each sample were found in dbSNP [15]. Below we also comment on the results obtained for 50 and 100 reads.

**Figure 1:**
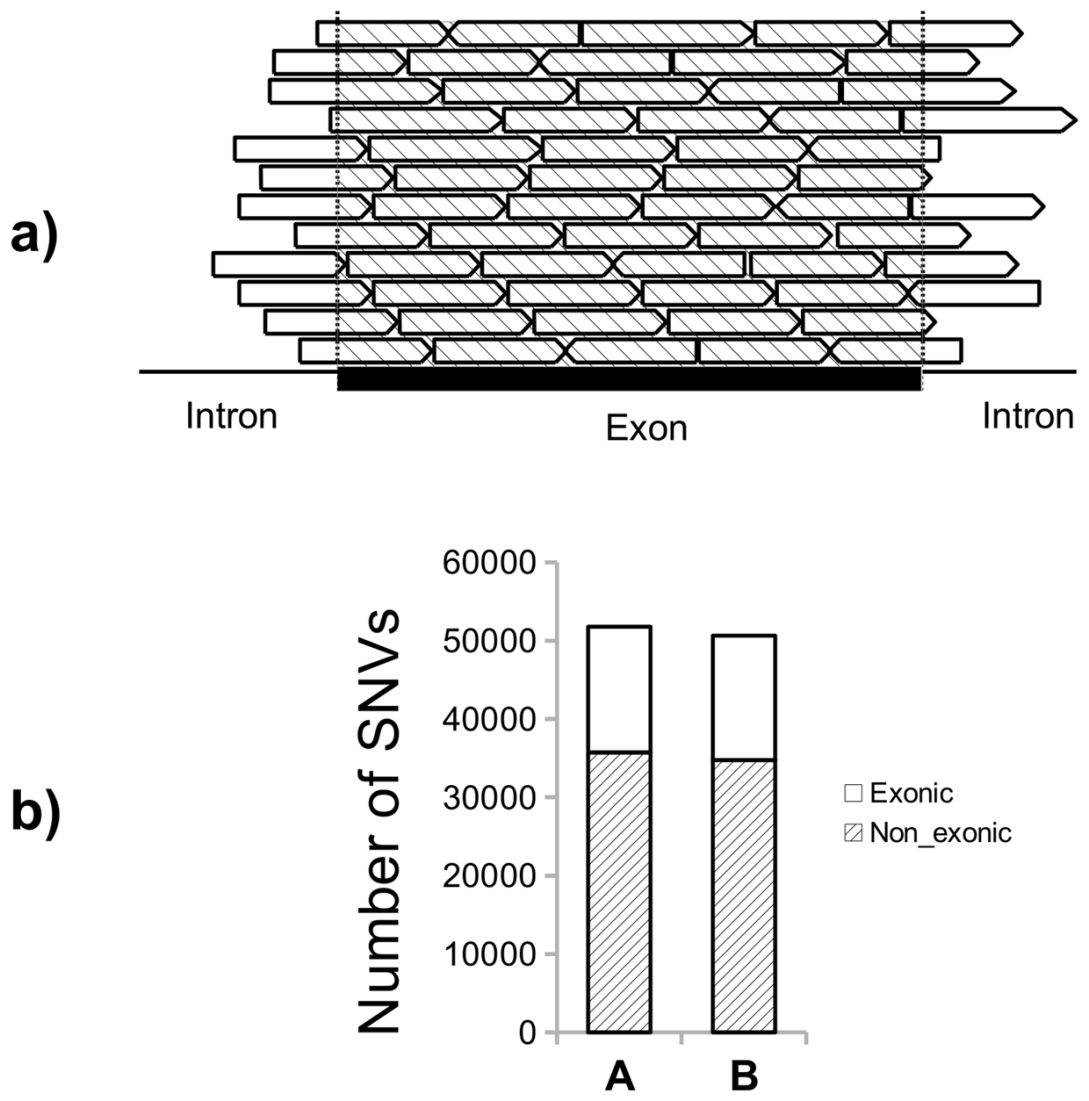
SNVs found in blood for individuals A and B. **a**) Scheme showing how flanking intronic sequences can be detected in exome sequencing. The capture of exonic reads includes intron regions at both ends of the exons. **b**) Total number of SNVs found in blood from subjects A and B. The annotation software of SNVs (see materials and methods) classified these variations as exonic and non-exonic. For the aims of this work, we considered only exonic variations.

Although exonic sequences were enriched (see methods), non-exonic ones located at both ends of exons were also mapped (see Figure 1a). Figure 1b shows the appearance of intronic and exonic SNVs. However, for the rest of the study we focused on the latter. Due to the differences in age of the two subjects (A and B), we can not rule out that some of the observed differences between those persons could be based in that differences of age. However, as indicated in the figure 1b the number of SNV are very similar in both individuals, which would suggest that most SNVs arise from differences during development.

**Figure 2:**
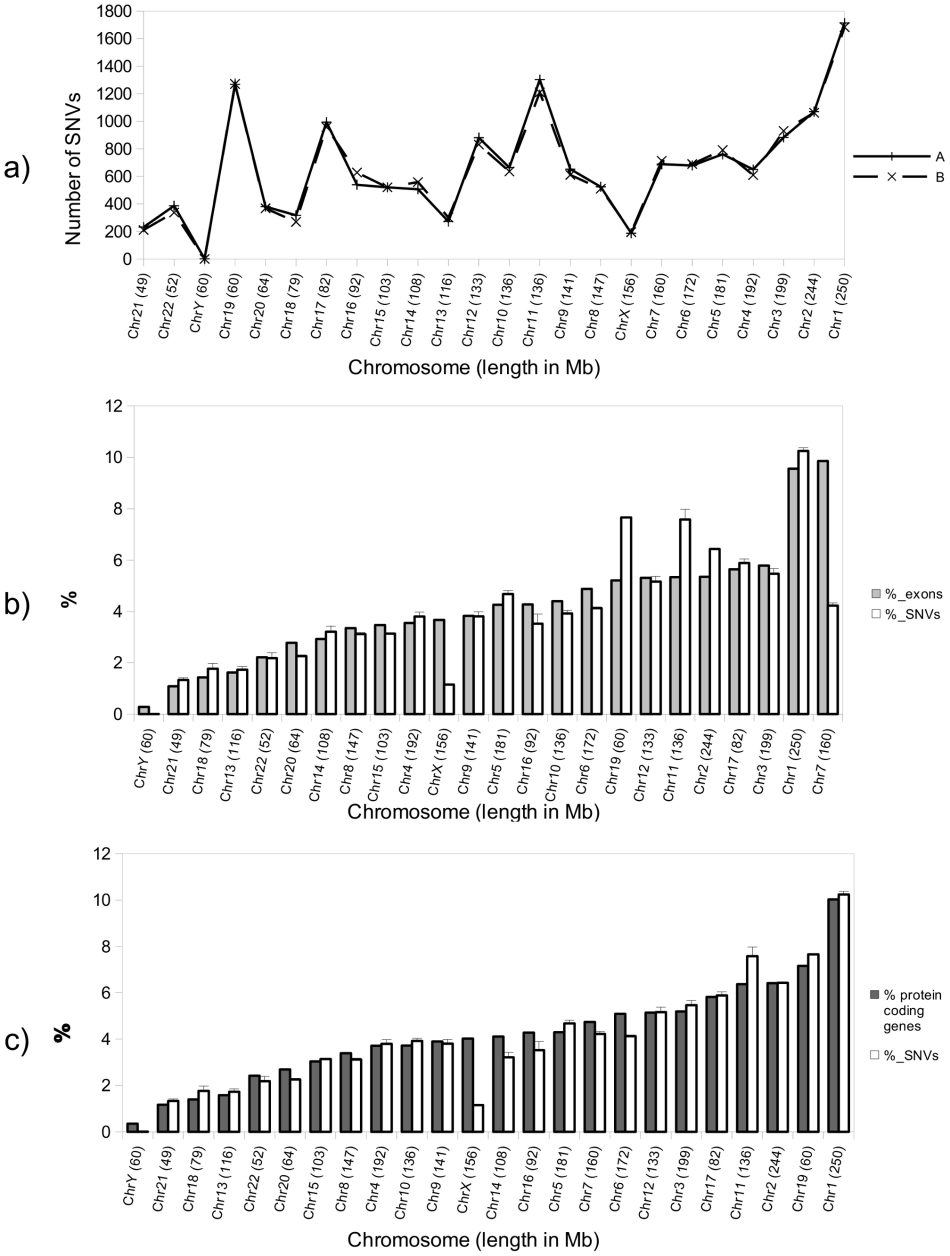
Number and distribution along the chromosomes of blood exonic SNVs. **a**) Total number of exonic SNVs found in blood for each chromosome for subjects A and B. The chromosomes are sorted by size in Megabases from low to high. No correlation is appreciated between the length of the chromosome and the number of SNVs found in each case. **b**) White bars represent the percentage of SNVs per chromosome with respect to the total SNVs found in blood for subjects A and B. Gray bars indicate the percentage of exons (with respect to the number of total exons in the human genome [21]) for each chromosome. Bars were sorted in this case from lower to higher number of exons/chromosome. There seems to be a certain correlation between the number of exons and the number of SNVs per chromosome, with some exceptions, mainly for chromosomes 7, 2, 11, 19 and X. Error bars indicate the standard deviation of the measurements. **c**) As in b), white bars show the average of the percentage of total SNVs in each chromosome for subjects A and B, but in this case gray bars indicate the percentage of protein-coding genes with respect to the total number in the human genome[21]. Bars were sorted from lower to higher number of protein-coding genes/chromosome. Error bars indicate the standard deviation of the measurements.

To test whether the number of SNVs in a specific chromosome correlates with chromosome length or number of exons present, we calculated the number of SNVs in each chromosome. No clear relationship between the number of SNVs per chromosome and chromosome size in Mb was detected (Figure 2a).

Figure 2b points to possible link between the percentage of exons (with respect to total exons in genome [20]) per chromosome and the percentage of SNVs (with respect to total exonic SNVs found in each sample) for each chromosome. However, some exceptions were detected, mainly for chromosomes 2, 11 and 19, for which the percentage of SNVs was higher than that of exons. In other cases, like chromosomes 7 and X, the percentage of SNVs was lower than would be expected. Moreover, a better correlation in the number of SNVs per chromosome was appreciated when these were compared with the percentage of protein-coding genes in each case (according to the data obtained from Ensembl [21]) (Figure 2c). For chromosome X, there was an obvious difference between the number of protein-coding genes and the SNVs found in these conditions. This observation is consistent with the genetic diversity of chromosome X being lower than that of autosomes [22].

### Similar number and chromosomal distribution of SNVs but different chromosomal distribution of hippocampus and blood-specific SNVs

**Figure 3:**
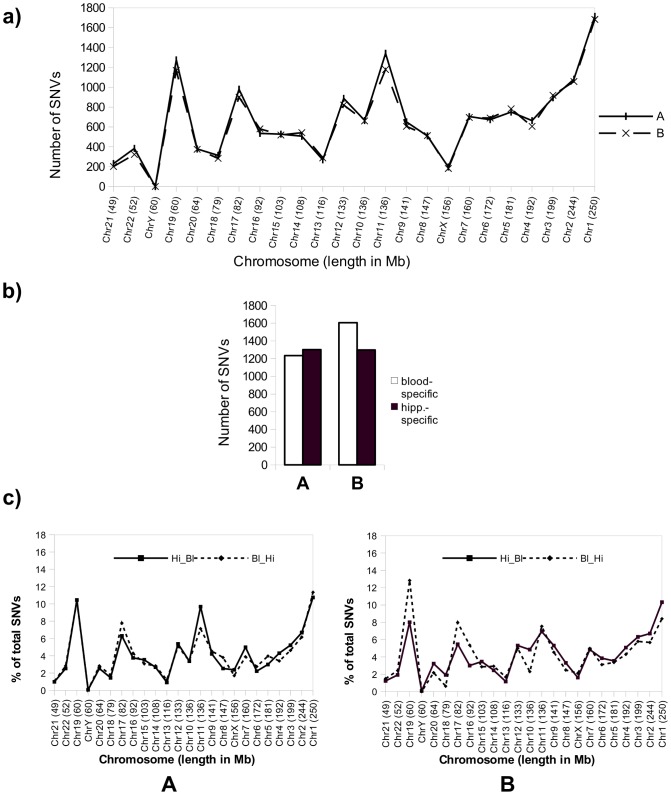
Number and distribution along the chromosomes of hippocampal- and blood-specific SNVs for individuals A and B. **a**) Chart showing the total number of exonic SNVs found in the hippocampus for each chromosome for subjects A and B. The chromosomes are sorted by size in Megabases from low to high. **b**) Chart showing the number of unique SNVs found in blood for subjects A and B with respect to hippocampus (white bars) and in hippocampus with respect to blood (black bars) for the same subjects. This number was obtained with a coverage of at least 20 reads per SNV (see methods). **c**) Distribution of the percentage (respect to total of SNVs) of unique SNVs present in chromosomes in blood for subjects A and B but not in hippocampus (dashed line, Bl_Hi) or in hippocampus but not in blood (continuous line, Hi_Bl).

To reveal possible differences in chromosomal distribution of SNVs in DNA sequences from two types of tissue from the same individual, we analyzed and compared the distribution of SNVs along all the chromosomes in exonic sequences in the hippocampus (Figure 3a) in individuals A and B. The number of hippocampal SNVs was found to be similar to that found in blood (see Figure 1A).

Figure 3b shows the total number of blood-specific SNVs (SNVs present in blood but not in the hippocampus of the same individual) and hippocampus-specific SNVs (present in the hippocampus but not in blood) for individuals A and B.

The screening of hippocampus- and blood-specific SNVs in all the chromosomes (Figure 3c) revealed that these variations did not occur in a random manner.

### Presence of a specific SNP at a given time when the exome was subjected to 20, 50 and 100 reads. Interpretation of the data

Sequence analyses were done using 3×10^−6^ g of DNA per sample. Assuming that each diploid male somatic cell in G1 phase of the cell cycle holds 6.4×10^−12^ g DNA [23], we can say that in ideal conditions we would have (3×10^−6^ g of DNA sample/6.4×10^−12^ g DNA by cell) ≃5×10^5^ cells per sample. Thus taking one DNA molecule, assuming all the cells in the sample to be identical, there would be 5×10^5^ identical DNA molecules of this type in the sample. However, if the sample contained more than one cellular type and only some of these cells had SNVs, there would be differences among the sequences of various types of cell, and the proportion of sequences containing these SNVs would correlate with the proportion of these cells in the whole tissue. If the variation were already in the germline, all the cells, regardless of their type, would have that SNV. Also, if the variation had taken place during development or early in life, the number of cells with that SNV would be higher than if the variation had occurred later during adulthood.

An estimate of the percentage of cells bearing a specific SNV can be inferred from the percentage of reads bearing that particular variation.

**Figure 4:**
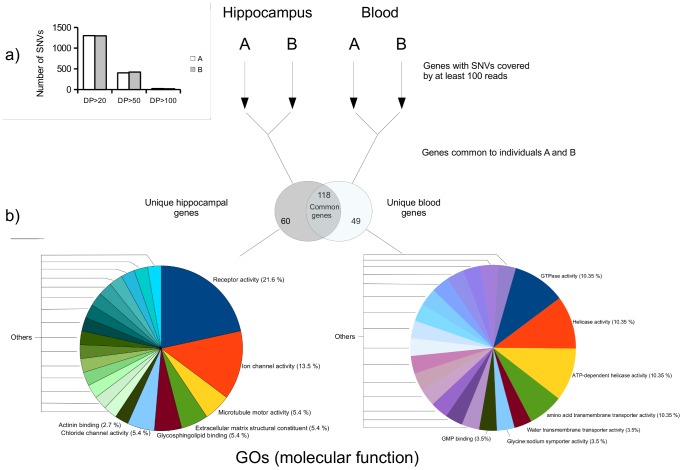
GO terms for genes with highly covered tissue-specific SNVs. **a**) (Inset) Number of hippocampus-specific SNVs (present in hippocampus, but not in blood) for subjects A and B, taking account the number of reads covering each SNV: at least 20 (DP20), 50 or more (DP50), and 100 or more (DP100). **b**) Analysis of GO terms for genes with highly covered SNVs. We selected lists of genes common to subjects A and B with at least one SNV in hippocampus and blood, covered by at least 100 reads, in order to test for differences in the annotations of the GOs of genes with SNVs tissue-specific. The genes with SNVs in hippocampus and blood covered by at least 100 reads were analyzed by examining genes exclusive to the hippocampus and those exclusive to blood. The annotations for molecular function GOs overrepresented in each list of hippocampus- (left) and blood- (right) specific genes are shown. Observe that the main annotations vary greatly for each tissue. There is overrepresentation of genes with SNVs in the hippocampus with functions of receptor activity (21.6%), ion channel activity (13.5%) and microtubule motor activity (5.4%), while for genes with highly covered SNVs in blood, the main molecular function annotations are GTPase activity (10.35%), Helicase activity (10.35%), and others.

Figure 4a (inset) compares the number of hippocampus-specific SNVs found in individuals A and B in function of the number of times the SNVs were read, 20 (DP20), 50 (DP50) or 100 (DP100). As expected, the number of SNVs decreased as the reads increased due to the heterogeneity of the sample. The high coverage of these SNVs was probably caused by the greater number of copies of the sequences containing these SNVs, these variants were probably formed early in development and they spread to their cellular lineage than those SNVs present after reading the sample 20 times.

SNVs sequenced after 100 reads could be validated by other sequencing techniques. Although we consider it difficult to validate SNVs detected only at lower reads, these variations may also be relevant because they may have arisen from changes occurring in late development.

### SNVs in tissue types are present in loci related to the physiology of the tissue

The presence of tissue-specific SNVs in a cell type could be caused by errors in DNA replication or reparation that occur during cell proliferation. When one of these SNVs is formed, it is spread to its cellular lineage. We thus postulated that some of these SNVs, originated early in development, may be common to many cells in the same tissue/organ.

Here we focused on the nature of the genes containing tissue-specific SNVs. We analyzed the molecular function of genes carrying these tissue-specific SNVs and selected only those genes in subjects A and B with SNVs with a coverage of 100 or more reads. We followed this strategy in order to select the SNVs that presumably originated earlier in the development. Two sets of genes with SNVs were formed: those common to the hippocampus (A and B samples), and those common to blood (A and B samples). Afterwards, we compared these two sets of genes and selected the genes bearing SNVs that were blood- or hippocampus-specific. The selected lists of genes were analyzed using a web-based software (GeneCodis [19]) to determine molecular functions that are significantly linked to genes of the lists. The GO terms obtained from the two lists differed greatly. For the genes with hippocampal SNVs, the software determined a significant enrichment in GO molecular functions including “receptor activity”, “ion channel activity” and “microtubule motor activity”, among others that could be related to neuronal activity. In contrast, for the blood SNVs, the functions determined were completely different (“GTPase activity”, “helicase activity”, “amino acid transmembrane activity”, “water transporter activity”, etc).

### Exonic DNA from different tissue types bears specific SNVs

**Figure 5:**
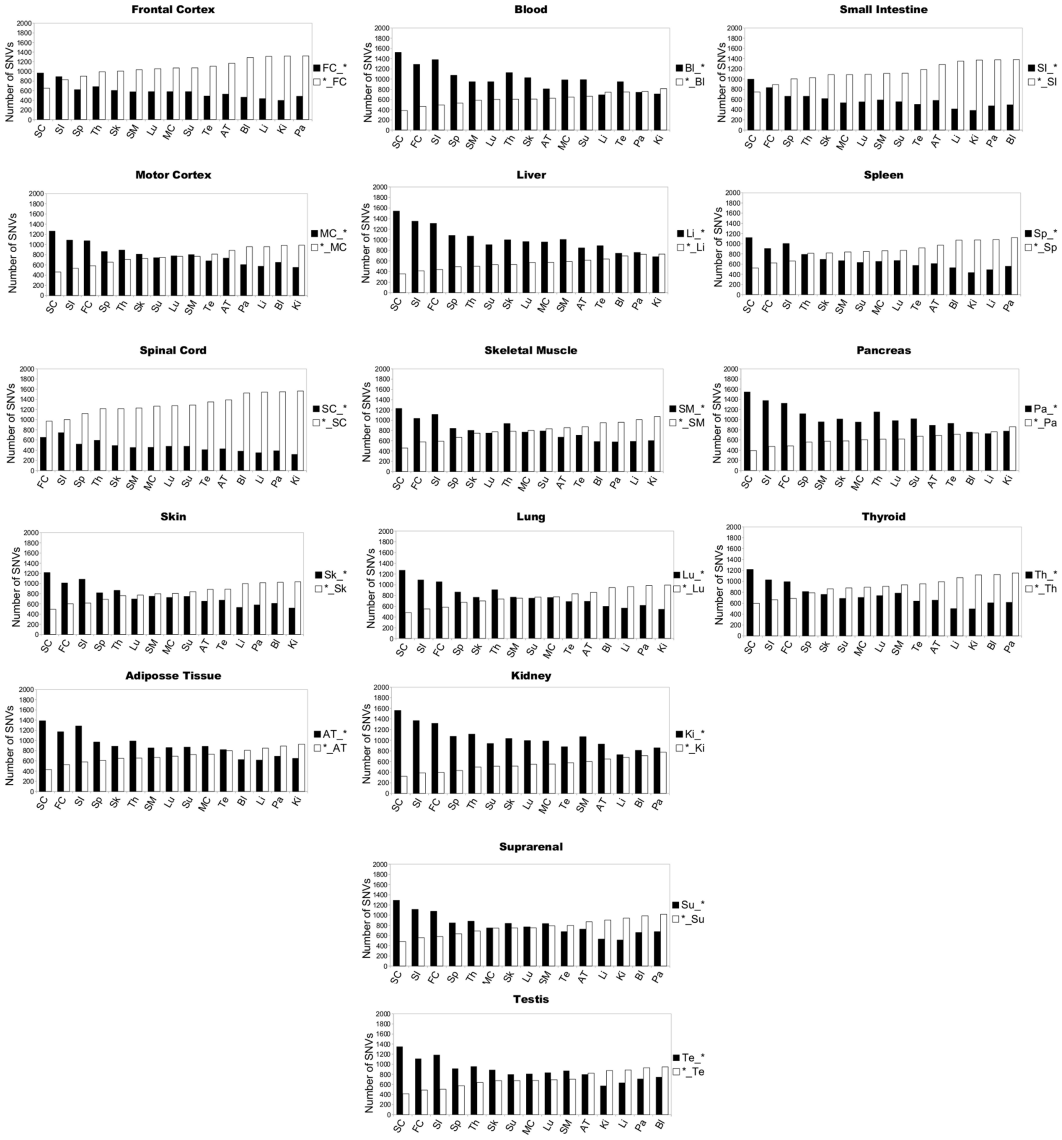
Pairwise comparison of tissue-specific SNVs. Tissue-specific SNVs from 16 tissues from subject C: Frontal Cortex (FC), Motor Cortex (MC), Spinal Cord (SC), Skin (Sk), Adipose Tissue (AT), Blood (Bl), Liver (Li), Skeletal Muscle (SM), Lung (Lu), Kidney (Ki), Suprarrenal Cortex (SC), Small Intestine (Si), Spleen (Sp), Pancreas (Pa) and Thyroid (Th). Each chart shows the number of unique SNVs found exclusively in each tissue. For example, for the Frontal Cortex, the black bars (FC_*) indicate the number of SNVs exclusive to this tissue with respect to Spinal Cord (SC), Small Intestine (SI) … etc; and white bars (*_FC) show the number of SNVs exclusive to the tissues with respect to the Frontal Cortex in each case. The bars were sorted from lower to higher in function of the results with respect to the white bars.

We performed exome sequencing of 16 tissues from the same individual (person C): adipose tissue (AT), blood (Bl), frontal cortex (FC), skin (Sk), testis (Te), skeletal muscle (SM), small intestine (SI), suprarrenal cortex (SC), spleen (Sp), pancreas (Pa), liver (Li), kidney (Ki), thyroid (Th), lung (Lu), spinal cord (SC), motor cortex (MC) and frontal cortex (FC) (Table 2). In this analysis, each tissue was compared pairwise with the rest of tissues at a depth of 20 reads per SNV (all these SNVs are supplied in file S1). Again, blood DNA was taken as a control (Figure 5). We found 14,446 SNVs common to all the tissues, including blood. These common SNVs may have been inherited, formed in the zygote or early in development. In addition, using pairwise comparison with the other tissues, a relative high number of SNVs were found to be present exclusively in blood DNA, thus raising the possibility that blood DNA is not an optimal tissue for genomic studies since it bears the largest number of somatic SNVs in adults.

**Table 2:** The tissues from subject C studied and their embryonic origin.

Tissue	Abreviation	Embryonic layer origin
Adipose Tissue	AT	ectoderm
Blood	Bl	mesoderm
Frontal cortex	FC	ectoderm
Kidney	Ki	mesoderm
Liver	Li	endoderm
Lung	Lu	mesoderm
Motor Cortex	MC	ectoderm
Pancreas	Pa	endoderm
Skeletal Muscle	SM	mesoderm
Skin	Sk	ectoderm
Small Intestine	SI	endoderm
Spinal Cord	SC	ectoderm
Spleen	Sp	mesoderm
Suprarrenal Cortex	SC	mesoderm
Testes	Te	mesoderm
Thyroid	Th	endoderm

Figure 5 also shows the presence of SNVs in a specific tissue (frontal cortex) that were not present in another (e.g., spinal cord). In addition, some SNVs were found in the spinal cord, but were absent in the frontal cortex. When this procedure was repeated by pairwise comparison in all the tissues, unique SNVs differed from tissue to tissue. Also, we found that these SNVs showed a tissue-specific distribution along the chromosomes. Figures S1-S6 show the distribution of tissue-specific SNVs and their position along the chromosomes when the frontal cortex was compared with three tissues of distinct embryonic origin: frontal cortex-spinal cord (Figures S1), frontal cortex-blood (Figure S3), and frontal cortex-pancreas (Figure S5). The figure also shows the distribution of the tissue-specific SNVs found when these three tissues were compared with the frontal cortex: spinal cord-frontal cortex (Figure S2), blood-frontal cortex (Figure S4) and pancreas-frontal cortex (Figure S6).

These findings suggest that some SNVs are tissue-specific in the same subject. We thus believe that while most SNVs are already present in germinal cells and are thus carried in every cell of the organism, others may originate during development (thus being shared by tissues of similar origin: ectoderm, mesoderm, endoderm) (Table 2). Finally, other SNVs might arise from somatic variations during adulthood and therefore they are present exclusively in a particular tissue.

### Number of total exonic SNVs along the chromosomes in different tissue types compared with those in blood DNA

**Figure 6:**
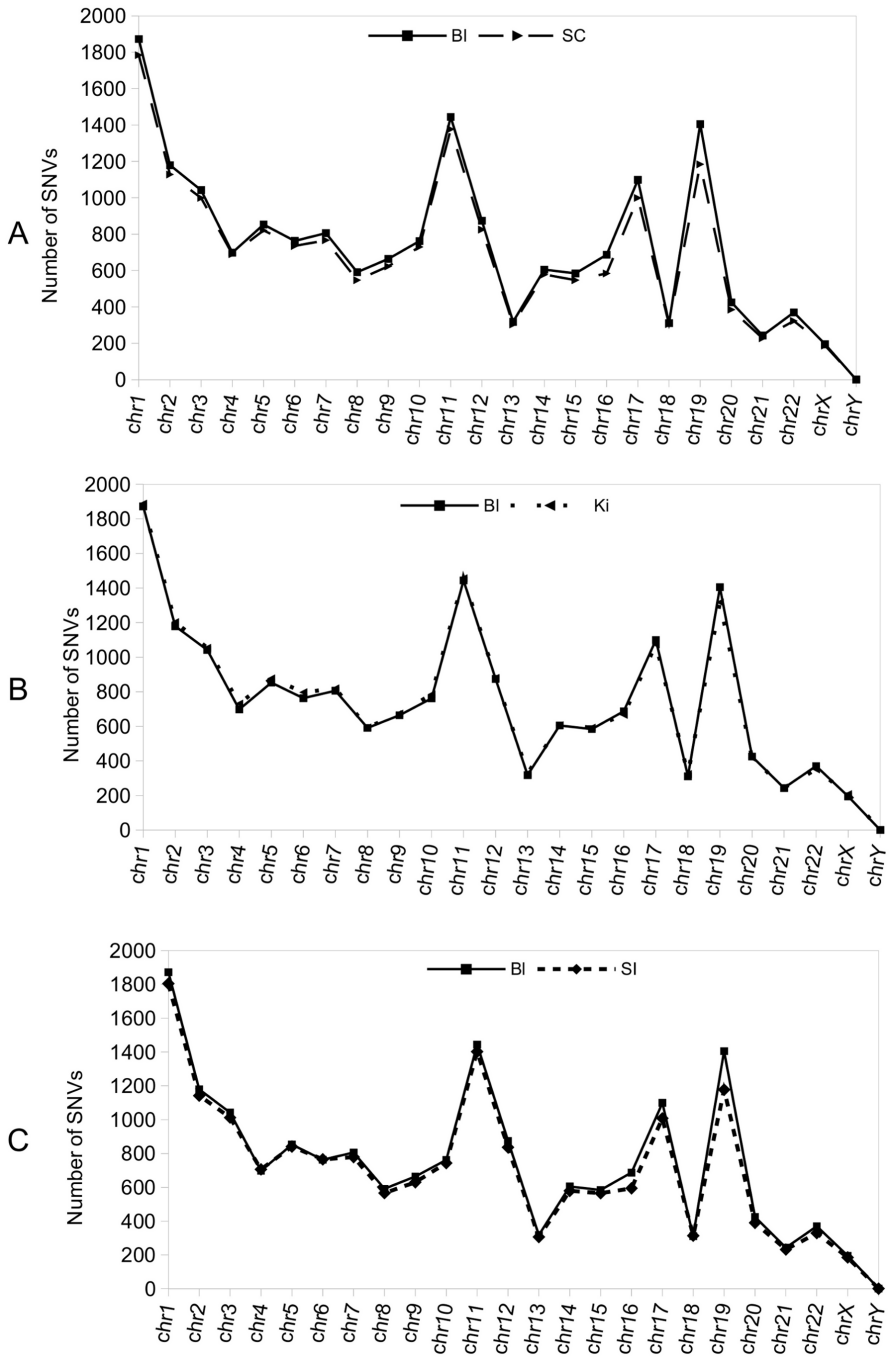
Number of total exonic SNVs found for each chromosome in tissues from subject C compared with blood. **a**) Exonic SNVs per chromosome found in a representative tissue of ectoderm origin, namely the spinal cord (SC), compared with those of blood (Bl). **b**) As in **a**) total blood exonic SNVs per chromosome are shown, but in this case they are compared with the number of SNVs present in a representative tissue of mesoderm origin, namely the kidney (Ki). **c**) Representation of the number of total exonic SNVs per chromosome found in a representative tissue of endoderm origin, namely small intestine (SI), compared with those of blood (Bl).

The presence of tissue-specific SNVs in the same individual implies a certain degree of genome heterogeneity, some of which may arise during development. Accordingly, the genetic variability between two tissues in the same subject would be higher or lower depending on whether they share the same embryonic origin. In order to test this hypothesis, we undertook a first approach by comparing the number of exonic SNVs per chromosome in blood DNA (mesoderm origin) against: spinal cord (ectoderm), kidney (mesoderm) and small intestine (endoderm). We detected similitude in the number of SNVs along the chromosomes in tissues of the same embryonic origin (blood and kidney, Figure 6b), compared with those of spinal cord and small intestine (Figure 6c, ectoderm and endoderm, respectively), and blood and spinal cord (endoderm and ectoderm, respectively). Major differences in the number of SNVs consistently appeared in certain chromosomes, especially in chromosomes 19, 17 and 16. Otherwise, as we suggested previously, the presence of SNVs in the same genes and tissues of different people could indicate that certain chromosomal locations are more susceptible to variations than others. The presence of many variations in certain chromosomes in the same individual supports this idea.

## Conclusions

Blood is a highly accessible tissue that can be collected in a non-invasive manner for the purposes of genetic analysis. Such analyses have shown that there is about 0.1% of inter-individual variation in sequences, which is caused by germinal and somatic mutations. Here we analyzed the number and distribution of SNVs in blood exonic sequences of two subjects using the Illumina method. A difference of about 0.1% in the SNVs of the genomic sequences of two subjects was found. Our results also revealed a similar number of total exonic SNVs for the two subjects, as well as a similar SNV distribution along chromosomes. The distribution of SNVs per chromosome did not appear to be determined by chromosome size or by the number of exons, but correlated well—with some exceptions—with the number of protein-coding genes present in the chromosomes. This observation supports the notion of a relationship between gene expression and SNV number. Comparison of tissues other than blood, like the hippocampus, showed the presence of tissue-specific variations. These SNVs were distributed along the chromosomes in a similar but different pattern to those found in blood. For every tissue studied, appears to be a common pattern in the distribution along the chromosomes of the number of SNVs, being: chr1-chr11-chr19, chr2 the list of sorted chromosomes with most SNVs (from high to low). Nevertheless, looking at the number of tissue-specific SNVs and their location, this pattern is different and it depends on the tissue tested.

Mosaicism in humans has been widely reported [2], [4], [24]–[26] and has been attributed to errors that occur during chromosome segregation or DNA replication and that are transmitted to the cellular lineage of the cell. Accordingly, some SNVs form at different stages of development. Those formed in germlines will be common to all cells of the organism; those generated during early embryonic stages will be common to this lineage; and those formed during late development or adulthood will be exclusive to a cell type or group of cells. Given the number of lineages present in a particular tissue, it is reasonable to assume that there will be SNVs common to all the cells in the tissue, while others will be shared by the same cell type or group of cells. Here we have used highly sensitive DNA sequencing technique to detect genomic variations. We propose that the higher the number of reads covering a SNV, the greater the number of DNA molecules having this variation and that these highly covered SNVs are formed early in development. To illustrate this, we analyzed the GO terms for molecular functions of genes containing highly covered SNVs (equal or more than 100 reads per SNV) in the hippocampus and blood. We observed an enrichment in GO annotations related to neural function in hippocampus-specific genes with highly covered SNVs. In contrast, blood-specific genes showed enrichment in other terms, these more related to metabolism.

The presence of tissue-specific SNVs, their particular distribution along the chromosomes, and the molecular function of genes with a higher coverage per SNV support the idea of mosaicism in tissues and the relationship between the type and number of SNVs and tissue cellular lineage.

In the second part, we analyzed unique SNVs in 16 tissues (of diverse embryonic origin) from the same individual. The comparison between tissues showed a different number and distribution of unique SNVs along the chromosomes when they were confronted between them by pairwise comparison. No clear relationship between the number of unique SNVs and the embryonic origin of the tissues, when compared against the rest by pairwise comparison, was detected. However, some differences were detected in the number of total SNVs per chromosome in tissues of distinct embryonic origin, while those from the same embryonic layer showed a similar SNV pattern and distribution along the chromosomes. The differences between tissues were specially marked in some particular chromosomes, mainly in 19, 17 and 16, thus supporting the notion that SNVs are more likely to form at certain locations.

At present, the results reported here can be obtained only with the method described. Future sequencing techniques may show increased sensitivity to detect SNVs present in a minority of cells in a tissue. In this regard, “traditional” methods, based on Sanger sequencing techniques, have the handicap that they are designed to sequence homogeneous samples. While recently reported methods of single-cell sequencing are promising for the detection of individual variations in a single cell, they are not fully developed, and the extensive PCR-based amplification used in this method might interfere with the resolution of this approach [27]. However, it should be tested whether new digital PCR techniques that could be now available, may solve this issue [28].

In summary, here we found tissue-specific differences in SNVs after testing 16 tissue types. The distribution of SNVs on the chromosomes indicates a specific signature for each tissue. The differences were found to be higher when comparing tissues of distinct embryonic origin.

## Supporting Information

Figures S1 Distribution along all the chromosomes of tissue-specific SNVs. Histograms showing the number of unique SNVs for all the chromosomes on the basis of tissue type. Each blue bar shows the number of tissue-specific SNVs per million of base pairs for the chromosomes **in the Frontal Cortex but not in the spinal cord** (FC-SC, **S1**).
(TIF)

Figure S2 Distribution along all the chromosomes of tissue-specific SNVs. Histograms showing the number of unique SNVs for all the chromosomes on the basis of tissue type. Each blue bar shows the number of tissue-specific SNVs per million of base pairs for the chromosomes **in** **the spinal cord but not in the Frontal Cortex** (SC_FC, **S2**).
(TIF)

Figure S3 Distribution along all the chromosomes of tissue-specific SNVs. Histograms showing the number of unique SNVs for all the chromosomes on the basis of tissue type. Each blue bar shows the number of tissue-specific SNVs per million of base pairs for the chromosomes **in the Frontal Cortex but not in blood** (FC_B, **S3**).
(TIF)

Figure S4 Distribution along all the chromosomes of tissue-specific SNVs. Histograms showing the number of unique SNVs for all the chromosomes on the basis of tissue type. Each blue bar shows the number of tissue-specific SNVs per million of base pairs for the chromosomes **in** **blood but not in the Frontal Cortex** (B_FC, **S4**).
(TIF)

Figure S5 Distribution along all the chromosomes of tissue-specific SNVs. Histograms showing the number of unique SNVs for all the chromosomes on the basis of tissue type. Each blue bar shows the number of tissue-specific SNVs per million of base pairs for the chromosomes **in the Frontal Cortex but not in pancreas** (FC_Pa, **S5**).
(TIF)

Figure S6 Distribution along all the chromosomes of tissue-specific SNVs. Histograms showing the number of unique SNVs for all the chromosomes on the basis of tissue type. Each blue bar shows the number of tissue-specific SNVs per million of base pairs for the chromosomes and **in pancreas but not in the Frontal Cortex** (Pa_FC, **S6**).
(TIF)

File S1 Tables containing all tissue-specific SNVs from individual C.
(XLS)
