# Supplementary figures and images for: Similarities and Differences between Exome Sequences Found in a Variety of Tissues from the Same Individual
Source: PLoS One. 2014 Jul 1;9(7):e101412. doi: 10.1371/journal.pone.0101412 (PMC4077829; doi:10.1371/journal.pone.0101412)

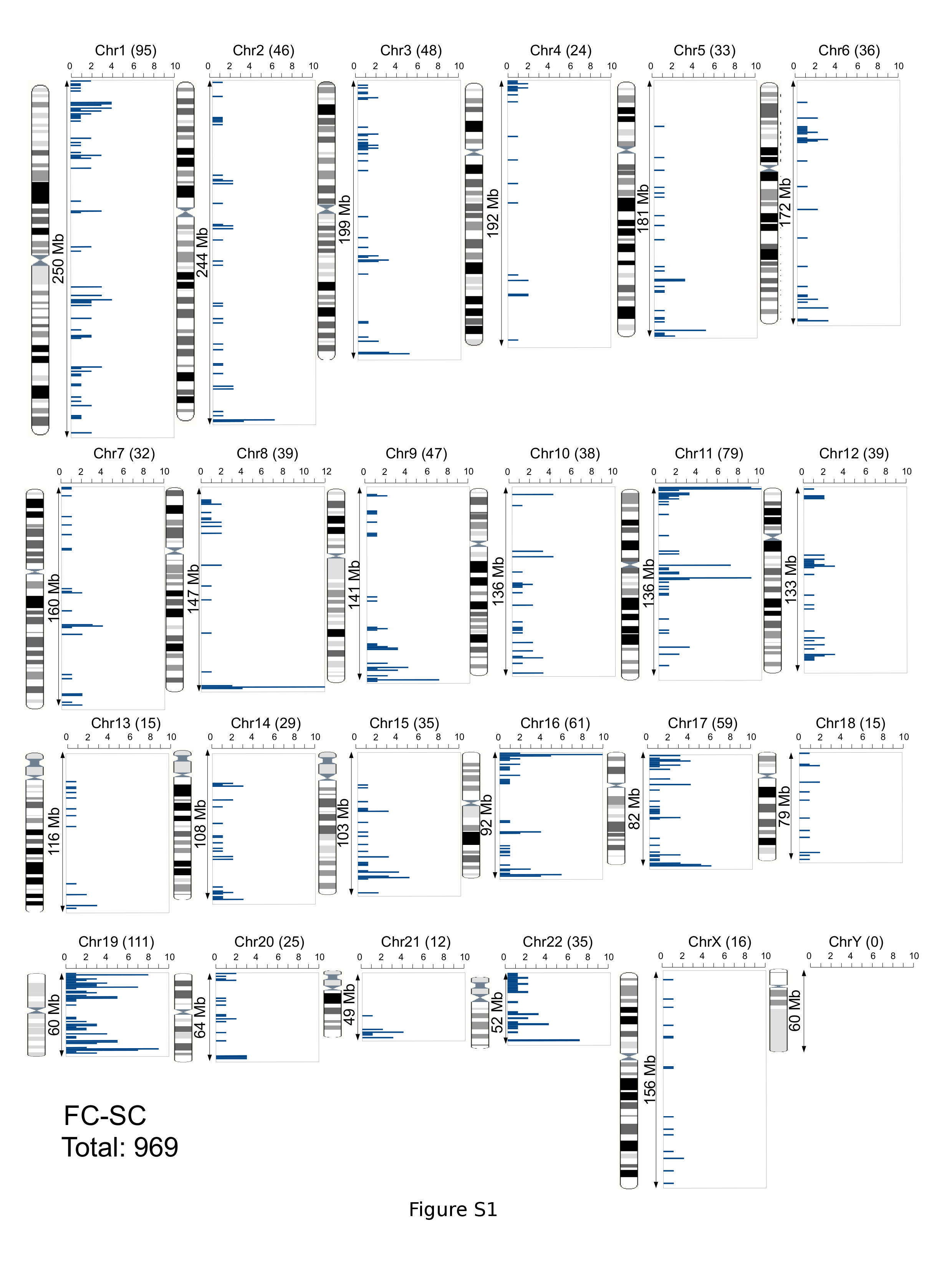

Supplement: Figures S1 — Distribution along all the chromosomes of tissue-specific SNVs. Histograms showing the number of unique SNVs for all the chromosomes on the basis of tissue type. Each blue bar shows the number of tissue-specific SNVs per million of base pairs for the chromosomes in the Frontal Cortex but not in the spinal cord (FC-SC, S1). (TIF) [file pone.0101412.s001.tif]

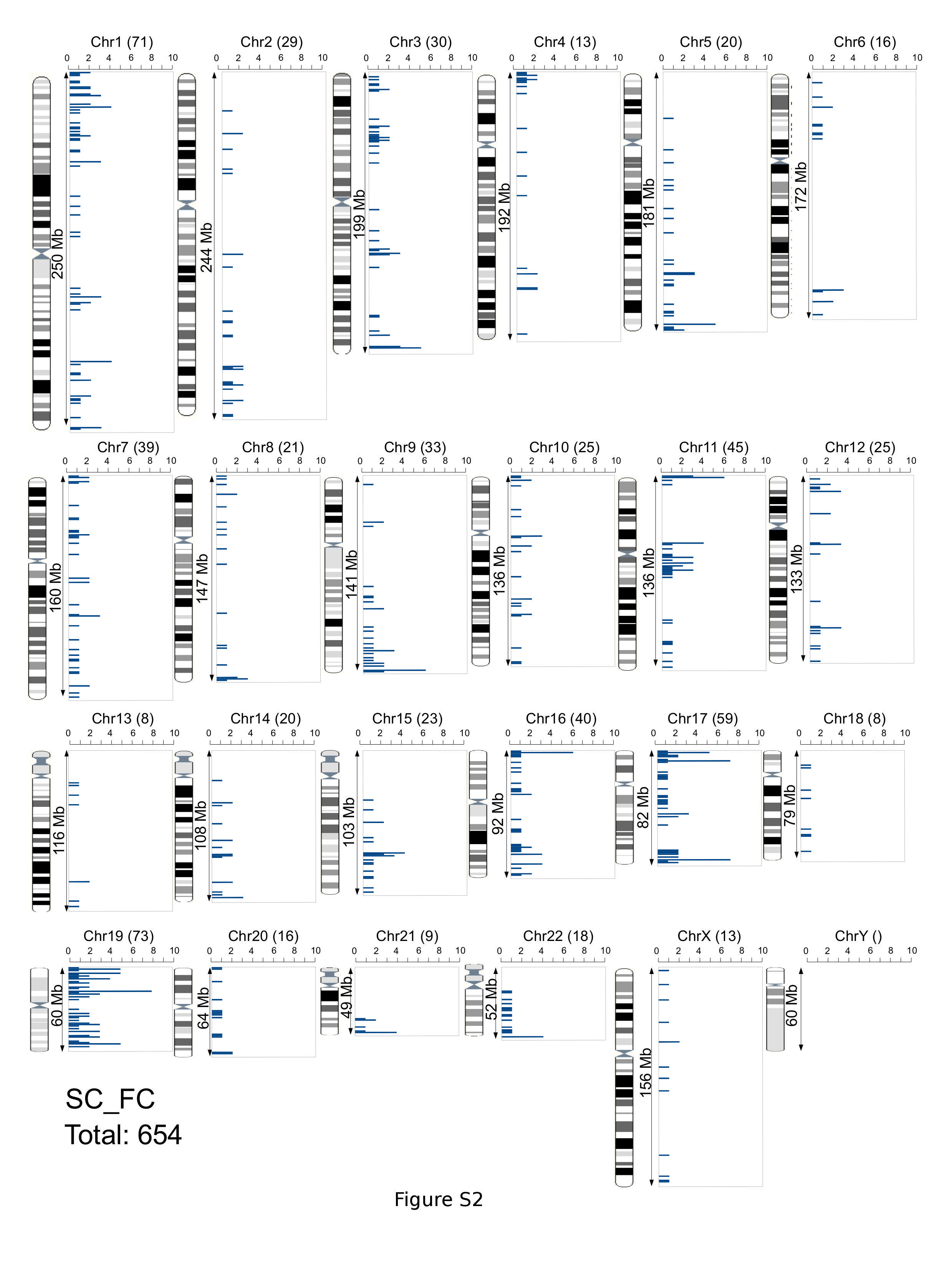

Supplement: Figure S2 — Distribution along all the chromosomes of tissue-specific SNVs. Histograms showing the number of unique SNVs for all the chromosomes on the basis of tissue type. Each blue bar shows the number of tissue-specific SNVs per million of base pairs for the chromosomes in the spinal cord but not in the Frontal Cortex (SC_FC, S2). (TIF) [file pone.0101412.s002.tif]

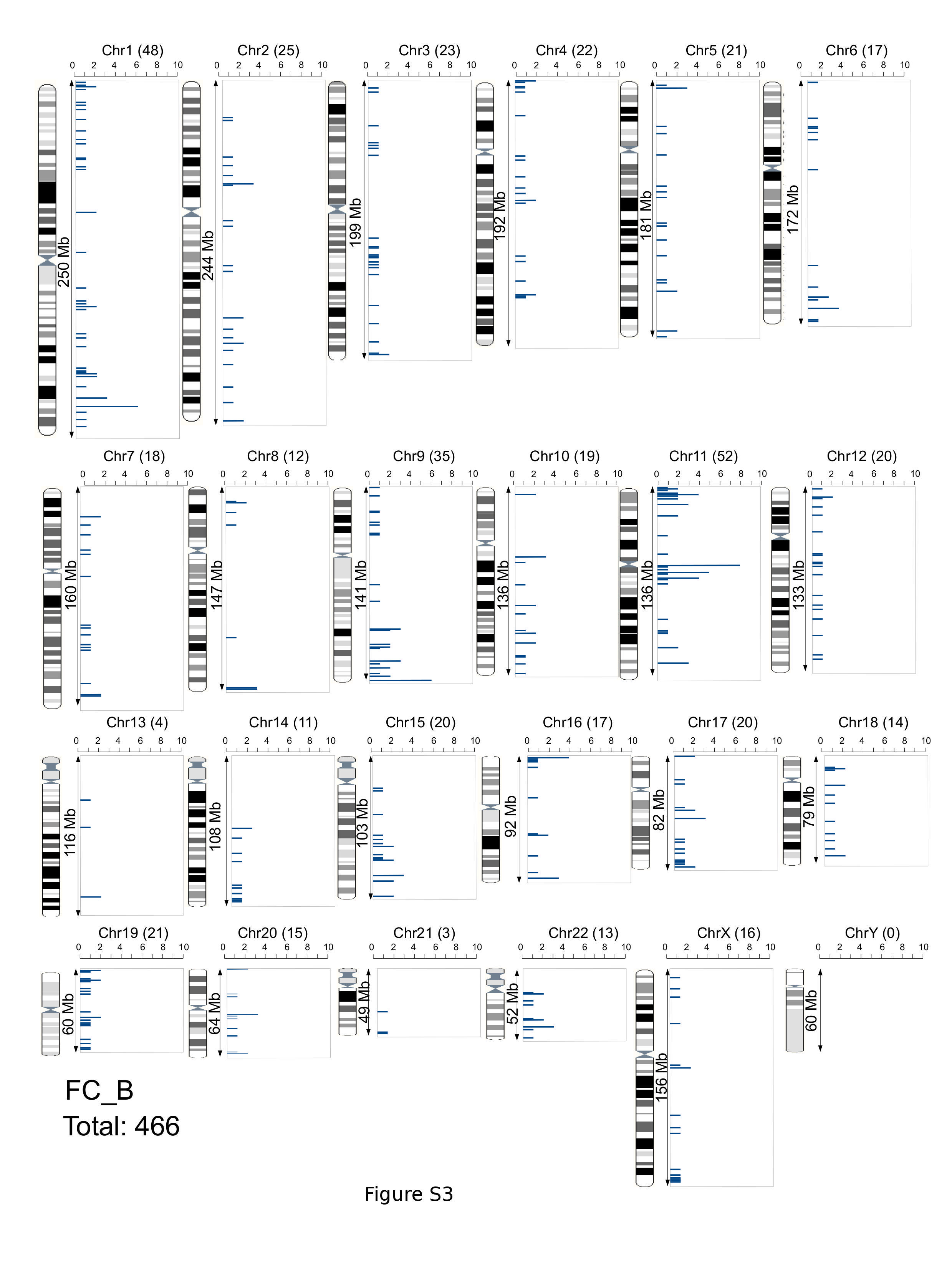

Supplement: Figure S3 — Distribution along all the chromosomes of tissue-specific SNVs. Histograms showing the number of unique SNVs for all the chromosomes on the basis of tissue type. Each blue bar shows the number of tissue-specific SNVs per million of base pairs for the chromosomes in the Frontal Cortex but not in blood (FC_B, S3). (TIF) [file pone.0101412.s003.tif]

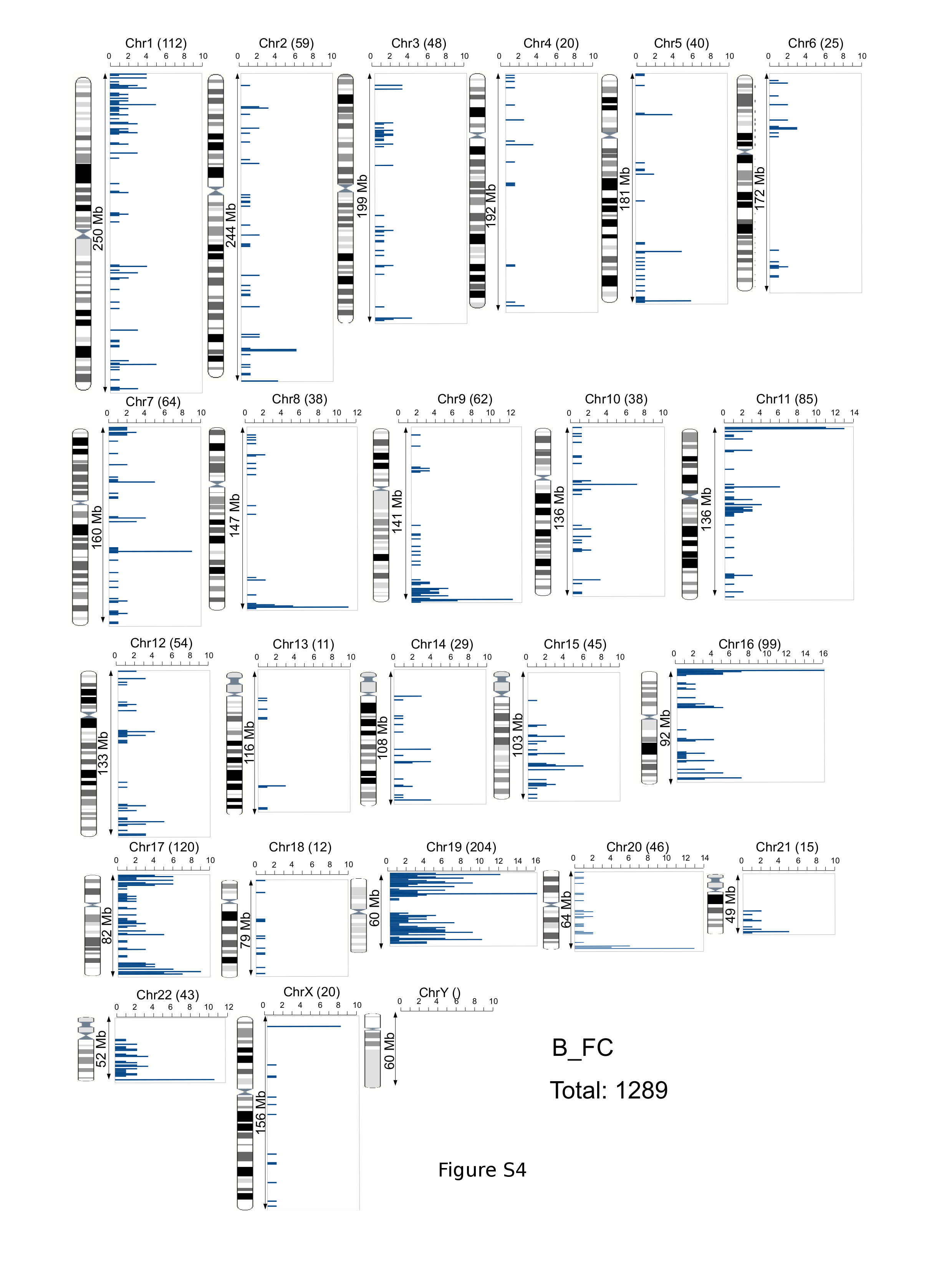

Supplement: Figure S4 — Distribution along all the chromosomes of tissue-specific SNVs. Histograms showing the number of unique SNVs for all the chromosomes on the basis of tissue type. Each blue bar shows the number of tissue-specific SNVs per million of base pairs for the chromosomes in blood but not in the Frontal Cortex (B_FC, S4). (TIF) [file pone.0101412.s004.tif]

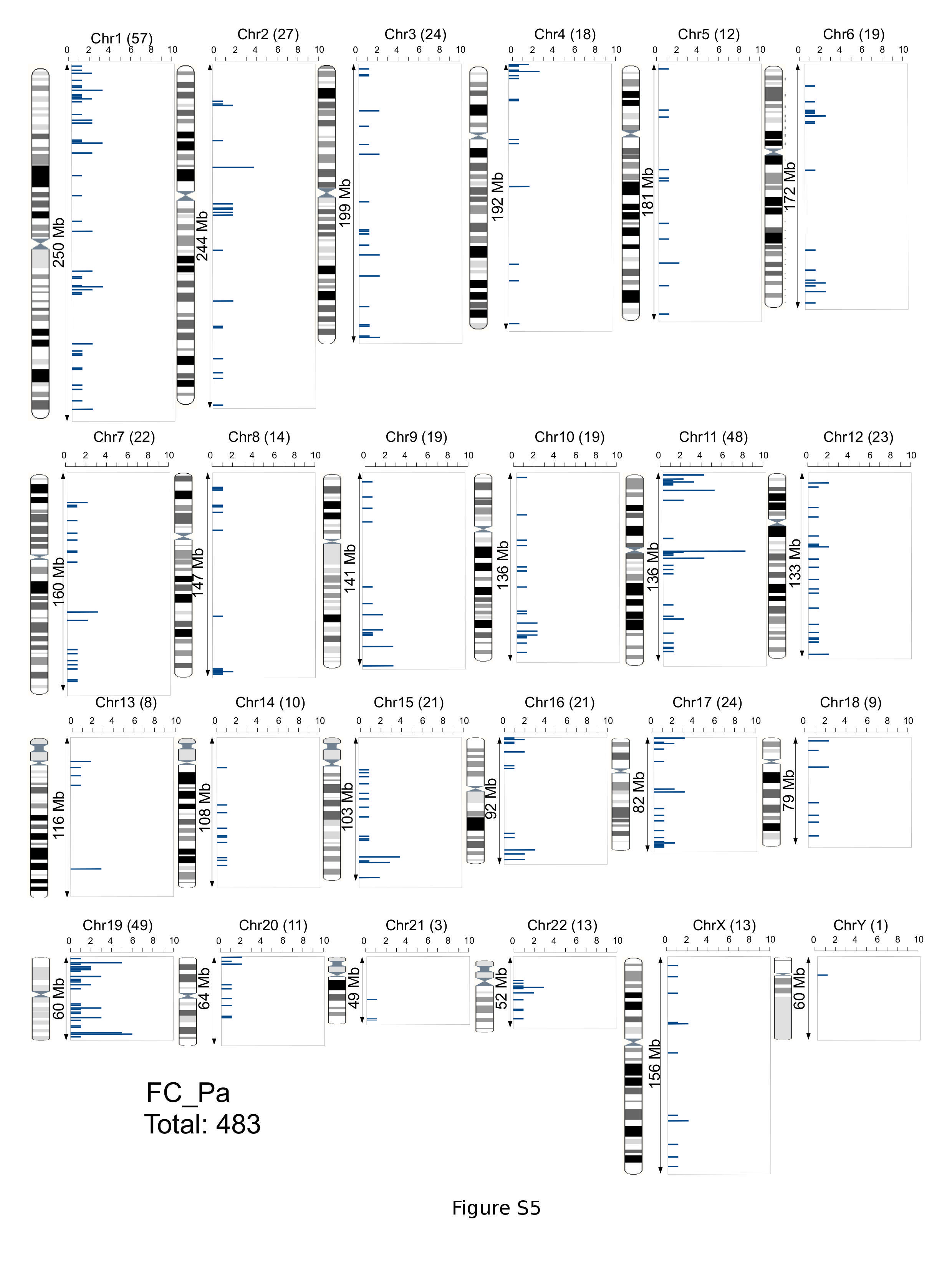

Supplement: Figure S5 — Distribution along all the chromosomes of tissue-specific SNVs. Histograms showing the number of unique SNVs for all the chromosomes on the basis of tissue type. Each blue bar shows the number of tissue-specific SNVs per million of base pairs for the chromosomes in the Frontal Cortex but not in pancreas (FC_Pa, S5). (TIF) [file pone.0101412.s005.tif]

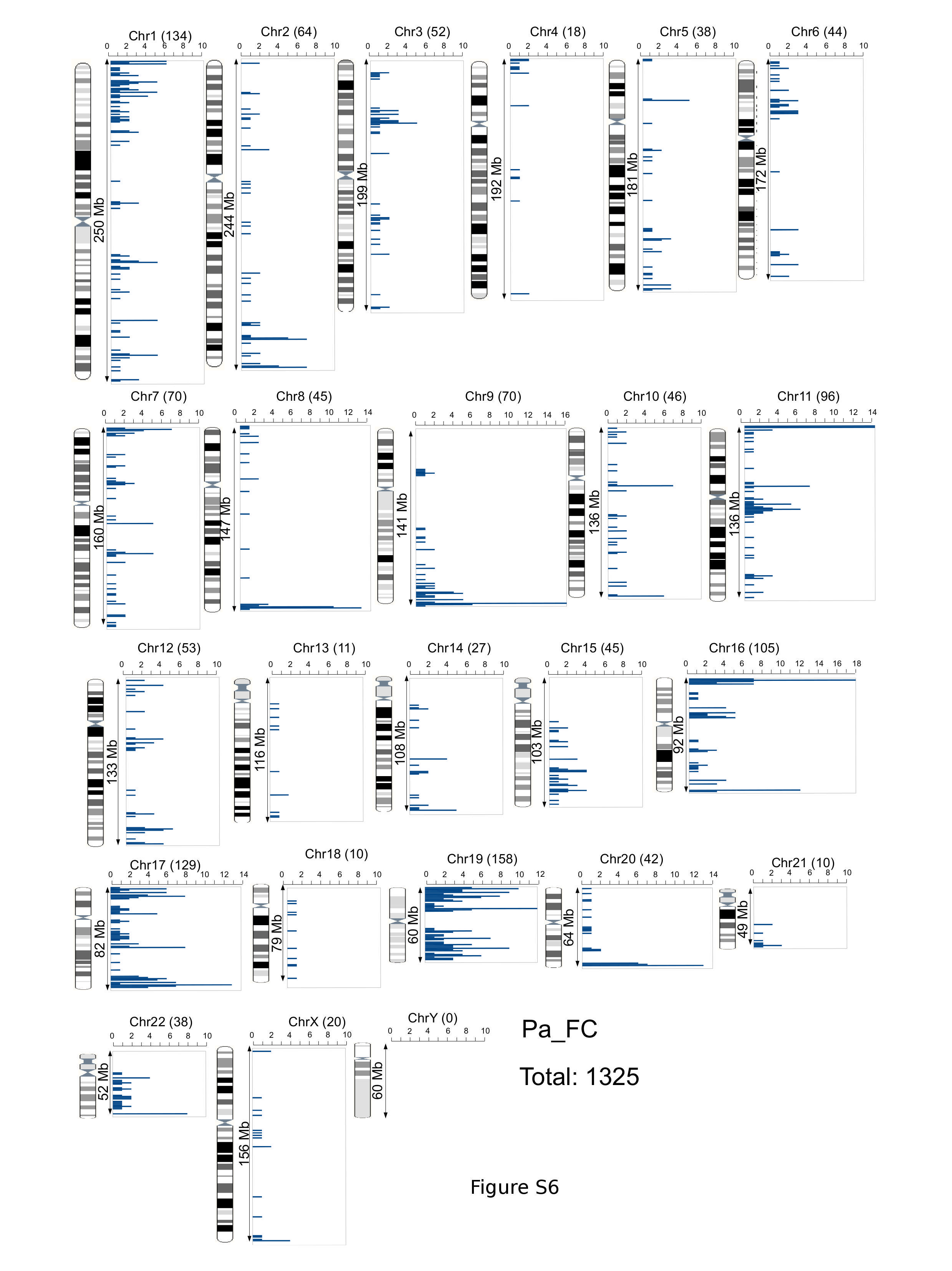

Supplement: Figure S6 — Distribution along all the chromosomes of tissue-specific SNVs. Histograms showing the number of unique SNVs for all the chromosomes on the basis of tissue type. Each blue bar shows the number of tissue-specific SNVs per million of base pairs for the chromosomes and in pancreas but not in the Frontal Cortex (Pa_FC, S6). (TIF) [file pone.0101412.s006.tif]
